# Performance of Short Food Questions to Assess Aspects of the Dietary Intake of Australian Children

**DOI:** 10.3390/nu5124822

**Published:** 2013-11-26

**Authors:** Gilly A. Hendrie, Malcolm D. Riley

**Affiliations:** Commonwealth Scientific Industrial Research Organisation (CSIRO), PO BOX 10041 Adelaide South Australia 5000, Australia; E-Mail: malcolm.riley@csiro.au

**Keywords:** diet, food habits, children, nutrition survey

## Abstract

Single dietary questions are used as a rapid method of monitoring diet. The aim of this investigation was to assess the performance of questions to measure population group intake compared to the mean of two 24-h recalls. Data from the Australian National Children’s Nutrition and Physical Activity Survey 2007 was used (*n* = 4487). Children reported their intake on three questions relating to usual serves of fruit, vegetables and type of milk. Age, gender and body weight status were assessed as modifiers of the relationship between methods. There was a stepwise increase in fruit and vegetable intake (*p* < 0.001) measured by recall when grouped by response category of the short question. By recall, fruit consumption decreased with age (*F* = 12.92, *p* < 0.001) but this trend was not detectable from the short question (*F* = 2.31, *p* = 0.075). The difference in fruit intake between methods was greatest for obese children. Almost 85% of children who consumed whole milk by short question consumed mainly whole fat milk by recall, but agreement was lower for other milk types. Saturated fat and volume of milk was highest in whole milk consumers. Ease of administration suggests that short questions, at least for some aspects of diet, are a useful method to monitor population intakes for children.

## 1. Introduction

There is a recognised need for brief methods to measure aspects of dietary intake that combine a low subject burden with the capacity for rapid reporting of population dietary intake, and to monitor population dietary change [1,2]. Short measurement tools are not expected to provide the richness of information that is achievable using more detailed dietary measurement methods, however they are likely to be useful in circumstances where efficient monitoring of key indicators of dietary intake is required. Intake of fruit and vegetables is generally lower than recommended [3], and programs to promote their consumption [4,5,6] are supported by monitoring population intake. While dairy foods are also under consumed by Australians [7], the common short question utilised in surveys seeks to determine the usual type of milk consumed. Australian dietary guidelines recommend the use of mainly reduced fat milk for children above the age of two years [8], and the type of milk used has been shown to indicate the percentage of total dietary energy intake contributed by saturated fat in children [2].

Short dietary questions used with children pose particular difficulties because food intake patterns and portion sizes generally differs between age groups, and the cognitive processes required to respond to dietary questions may also vary with age. An assessment of the validity of three short dietary questions applied to children in a national survey of Australian children [9] was recently reported by the Australian Institute of Health and Welfare [2]. Children’s response to short questions about usual fruit and vegetable intake, and milk type, were compared to estimates from 24-h recalls. However, it may be unrealistic to expect the same short question to perform equally well across the population; therefore, a more detailed investigation is warranted to provide further insight into the performance of the questions.

The aim of this investigation is to extend the evaluation of the three short dietary questions by considering how the question response is influenced by the frequency of intake and the stated serve size, and whether the performance of the question differs by children’s gender, age and weight status.

## 2. Experimental Section

The Australian National Children’s Nutrition and Physical Activity Survey 2007 included measurement of weight, height and food intakes in a nationally representative sample of 4487 children aged 2–16 years. A household quota sampling scheme from random clusters of postcodes resulted in children being selected proportional to the age and sex structure in each state or territory. The final response rate was 40%. A detailed description of the sample characteristics are presented elsewhere [9].

### 2.1. Dietary Intake Assessment Methods

Detailed dietary intake information was obtained on two days for each child using a three pass 24-h dietary recall administered by computer assisted personal interview (CAPI) followed by computer assisted telephone interview (CATI) between 7 and 21 days later. The main care giver was the primary source of dietary intake information if children were aged 2–8 years, and children aged 9–16 years reported their own intake. Food intake data were converted to nutrient intakes using an Australian food composition database (AUSNUT 2007).

Participants also responded to short dietary questions as part of the CAPI. The wording of the three short questions is shown in Table 1. Interviewers had the option to show a half cup measure to describe a serve of fruit and vegetables if necessary. Responses to the short questions were compared to grouped intake data from the dietary recalls. Responses to the fruit and vegetable short questions were recorded as zero for “doesn’t eat” fruit/vegetables, 0.5 serves for “less than one serve”, six for “six or more”, and all other values were as reported.

**Table 1:** Short questions used in the 2007 Australian National Children’s Nutrition and Physical Activity Survey and their response categories.

Short question	Response category
1. How many serves of fruit do (you/your child) usually eat each day? One serve is equal to half a cup. INTERVIEWER NOTE: Show food prompt if necessary ^1^.	Doesn’t eat fruit Less than one serve One serve Two serves Three serves Four serves Five serves Six or more serves
2. How many serves of vegetables do [you/your child] usually eat each day? One serve is equal to half a cup. INTERVIEWER NOTE: Show food prompt if necessary ^1^.	Doesn’t eat fruit Less than one serve One serve Two serves Three serves Four serves Five serves Six or more serves
3. What is the main type of milk that (you/your child) usually use?	Whole/full cream Low/reduced fat Skim Evaporated or sweetened condensed Soy milk None of the above Does not drink milk Don’t know

^1^ Interviewer instruction manual states “Show the 1/2 cup measure to describe serve if necessary”.

### 2.2. Statistical Analysis

Foods included in the fruit (±juice) and vegetables food groups were extracted from the 24-h recall data based on the 5 digit level codes (minor food group) and where necessary the 8 digit level (representing individual foods). The amounts of fruit and vegetables (in grams) were converted to 75 g serves based on the description given in the short question. The intake of fruit was also calculated as 150 g serves because this is consistent with the suggested serve size in the national food based recommendations for Australians (The Australian Guide to Healthy Eating [10]). The mean of two days of intake was calculated.

The mean frequency of fruit and vegetable consumption from the dietary recalls was also calculated by summing the entries for fruit or vegetables for each day.

Responses to short questions were plotted against mean grouped intake data from the dietary recalls, and compared using analysis of variance. Mean population intake and frequency of intake of fruit, fruit and 100% juice and vegetables (in serves) were calculated as the mean of two days from the 24-h recalls. The difference in estimated intake between the short question and 24-h recall was calculated for each individual with a positive difference value representing a higher estimation of intake by the short question.

The main type of milk used was derived from the recall data for each individual as the type of milk that contributed the greatest percentage of the total volume consumed, and as the highest contributor to the total occasions of milk consumed. Both methods of determining “main type of milk” from the dietary recall were compared to the grouped response from the short question on milk type.

Children’s gender, age and body weight status were assessed as potential modifiers of the relationship between the measurement methods. For children, BMI (body mass index) was converted to a z-score and adjusted for age and sex by using the least mean squares method [11]. Because of the lack of Australian data, calculations were based on the US Centres for Disease Control reference data provided as a computer program [11]. Children’s BMI z-score was classified by using the International Obesity Task Force definition [12].

Differences between gender, age, and weight status subgroups of the population were assessed using analysis of variance, and the mean difference between dietary measurement methods estimated using paired sample *t*-tests. Population weights were applied to all analyses to account for the non-proportional sampling scheme of the survey. These weights were based on the age, gender and region distributions of the 2006 Census of Population and Housing. The population weights were rescaled to the size of the sample for inferential statistics and to calculate 95% confidence limits for the mean estimates. Statistical significance was taken to be *p* < 0.05. All analyses were conducted using IBM SPSS Statistics 20 (SPSS Inc., Chicago, IL, USA).

## 3. Results

**Figure 1:**
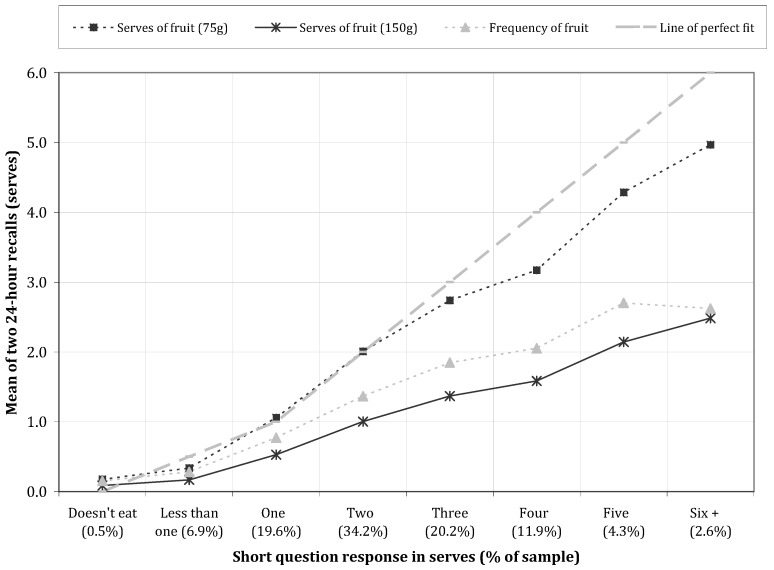
Estimated fruit intake from the 24-h recalls by reported intake on the short question.

**Figure 2:**
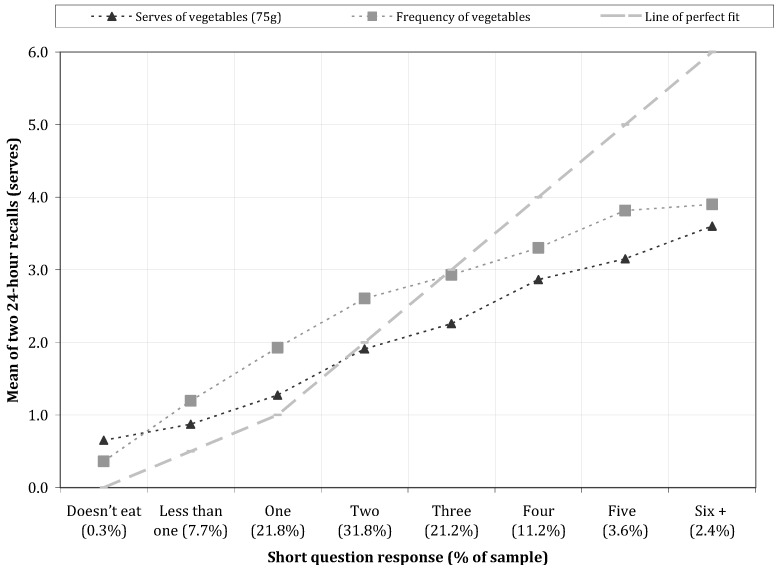
Estimated vegetable intake from the 24-h recalls by reported intake on the short question.

There was a stepwise increase in the mean fruit (*p* < 0.001, Figure 1) and vegetables (*p* < 0.001, Figure 2) intake measured by dietary recall when grouped by response category of the respective short question.

Frequency of consumption closely tracked the average number of serves of fruit and vegetables consumed when a 75 g serve size was used for vegetables and a 150 g serve size used for fruit.

The short question provided a markedly higher estimate of fruit and vegetable intake than the grouped recall data when the response for fruit intake was four serves a day or more, and for vegetables intake, was three serves a day or more. This accounted for 18.8% and 38.4% of responses, respectively.

Mean consumption of 100% fruit juice did not vary across levels of reported fruit intake by short question (average = 112 mL, *F* = 0.605, *p* = 0.752), including the “does not eat fruit” category. When measured by dietary recalls, mean fruit intake decreased with age group for both boys and girls (Table 2, linear trend *F* = 12.92, *p* < 0.001), while vegetable intake increased with age group (linear trend *F* = 20.61, *p* < 0.001). The same trend was not detectable when fruit intake was measured by short question (*F* = 2.31, *p* = 0.075), but the trend was observed for vegetable intake measured by short question (*F* = 72.91, *p* < 0.001).

**Table 2:** Estimated mean intakes of fruit, fruit and juice, and vegetables (in serves) from the short questions and mean of two 24-h recalls, by gender and age group ^1^.

Gender/age group		Short question	Mean of two 24-h recalls		Short question	Mean of two 24-h recalls
		Fruit	Fruit	Fruit and juice ^2^	Vegetables ^3^	Vegetables
	*n*	Mean (95% CI)	Mean (95% CI)	Mean (95% CI)	Mean (95% CI)	Mean (95% CI)
Males						
2–3 years	550	2.4 (2.3–2.5)	2.4 (2.2–2.6)	3.6 (3.3–3.9)	1.6 (1.5–1.7)	1.4 (1.2–1.5)
4–8 years	613	2.4 (2.3–2.5)	2.5 (2.4–2.6)	4.1 (3.9–4.3)	2.1 (2.0–2.1)	1.7 (1.6–1.8)
9–13 years	525	2.4 (2.3–2.5)	2.0 (1.9–2.2)	3.7 (3.4–3.9)	2.5 (2.4–2.6)	2.1 (2.0–2.2)
14–16 years	561	2.2 (2.0–2.3)	1.7 (1.5–1.9)	3.5 (3.1–3.8)	2.8 (2.7–3.0)	2.4 (2.2–2.7)
Females						
2–3 years	521	2.3 (2.2–2.4)	2.3 (2.1–2.5)	3.3 (3.1–3.6)	1.6 (1.5–1.7)	1.5 (1.3–1.6)
4–8 years	603	2.4 (2.3–2.5)	2.4 (2.3–2.5)	3.6 (3.4–3.7)	2.0 (2.0–2.1)	1.7 (1.6–1.9)
9–13 years	585	2.4 (2.3–2.5)	2.1 (1.9–2.2)	3.5 (3.4–3.7)	2.5 (2.4–2.6)	2.2 (2.0–2.4)
14–16 years	529	2.3 (2.1–2.4)	1.8 (1.6–1.9)	3.5 (3.3–3.8)	2.8 (2.7–2.9)	2.3 (2.1–2.5)

^1^ Population weighting applied. ^2^ A serve of fruit is equal to 75 g. “Fruit and juice” includes 100% fruit juice and no maximum limit on juice consumption. ^3^ A serve of vegetables is equal to 75 g. Vegetables includes potatoes and legumes, but not fried potato foods (chips or crisps).

Estimated fruit intakes were similar between methods for males and females aged 2–8 years, however for children in age groups 9–13 years and 14–16 years significantly higher intakes were reported by the short question compared to the dietary recalls (0.3–0.5 serves, *p* < 0.001 Table 3).

**Table 3:** Difference in reported fruit and vegetables intake (in serves) ^1^ between short question and mean of two 24-h recalls, by age group and child gender ^2,3^.

	Child gender	
Age group	Male	Female ^3^
	Mean (95%CI)	Mean (95%CI)
Fruit		
2–3 years	−0.01 (−0.18–0.15) ^a^	0.00 (−0.18–0.17) ^a^
4–8 years	−0.09 (−0.20–0.02) ^a^	0.04 (−0.07–0.15) ^a^
9–13 years	0.32 (0.19–0.45)	0.36 (0.23–0.48)
14–16 years	0.48 (0.32–0.64)	0.51 (0.36–0.66)
Difference between age groups ^4^	<0.001	<0.001
Vegetables		
2–3 years	0.18 (0.05–0.32)	0.12 (−0.03–0.27) ^b^
4–8 years	0.39 (0.29–0.50)	0.29 (0.18–0.40)
9–13 years	0.40 (0.27–0.54)	0.34 (0.19–0.49)
14–16 years	0.38 (0.16–0.59)	0.49 (0.31–0.67)
Difference between age groups ^4^	ns	0.047

^1^ For fruit and vegetables a serve is equal to 75 g. ^2^ Adjusted population weight for inferential statistics applied. ^3^ Positive values represent higher reported intake by short question compared to 24-h recalls. ^4^ Difference between males and females were statistically non significant. ^5^ One Way Analysis of Variance. ^a^ significantly different to 9–13 years and 14–16 years, ^b^ significantly different to 14–16 years.

Overestimation of vegetable intake by the short question compared to 24-h recall was apparent for all age groups except females aged 2–3 years. The difference in estimates of fruit and vegetable intake between methods was similar for males and females, increased with age for fruit intake (*p* < 0.001) but the age group trend for vegetable intake was not consistent between males and females (Table 3).

Overall, obese children (*n* = 247, 5.5% of the sample) reported a lower mean fruit intake compared to normal weight children (*n* = 3267, 72.8%) in response to the short question (−0.26 serves, *p* = 0.012) and by 24-h recall (−0.60 serves, *p* < 0.001). The difference in estimate of intake between methods differed significantly by children’s weight status for fruit (*F* = 3.34, *p* = 0.018) but not vegetable intake. The difference in estimated fruit intake between methods was greater for obese children (0.5 serves greater by short question) than normal weight children (0.2 serves, *p* = 0.010).

Almost 85% of children who reported their main milk type was whole milk by the short question consumed mostly whole milk as evidenced by the recalls, but the agreement was significantly lower for those who responded that low/reduced fat milk was their usual type (71.0%, *p* < 0.001) or that skim milk was their usual type (53.9%, *p* < 0.001). Agreement was similar when usual milk type by recall was calculated using frequency of consumption rather than volume of consumption, but slightly lower for cow’s milk categories when flavoured milk was included (Table 4).

**Table 4:** Main type of milk usually used: percent agreement (%) of short question response with two 24-h recalls, and percentage of energy from saturated fat (%) and volume consumed (mL) by short question response.

	Short question response ^1^					
	Whole/full cream (*n* = 2842, 63.3%)	Low/reduced fat (*n* = 1143, 25.5%)	Skim (*n* = 244, 5.4%)	Soy milk (*n* = 117, 2.6%)	Does not drink milk (*n* = 75, 1.7%)
	Percentage agreement					
By dominant proportion of volume—plain white milk only ^2^	84.8	71.0	53.9	63.6	66.7
By dominant proportion of volume—plain and flavoured milk ^2^	83.5	66.7	45.7	72.2	63.0
By highest frequency of use	81.4	71.5	53.6	64.1	66.7
Didn’t drink any milk on either day of the survey ^2^	9.8	12.3	16.6	18.8	66.7
Mean (95% Confidence interval)						
Overall	14.3 (14.2–14.5)	12.7 (12.6–12.9)	12.6 (12.1–13.1)	10.5 (9.8–11.1)	12.4 (11.5–13.2)
Child aged 2–8 years	14.6 (14.5–14.8)	12.5 (12.3–12.8)	12.4 (11.5–13.4)	10.1 (9.3–11.0)	11.8 (10.5–13.0)
Child aged 9–16 years	14.1 (13.9–14.2)	12.9 (12.6–13.1)	12.6 (12.1–13.2)	10.8 (9.8–11.8)	13.0 (10.5–13.0)
Plain milk	635.5 (618.2–652.7)	542.0 (518.7–565.3)	451.5 (398.8–504.1)	517.6 (433.6–601.5)	227.5 (95.9–359.0)
Plain and flavoured milk	704.1 (685.6–722.6)	611.5 (587.0–636.1)	560.0 (500.3–619.6)	592.3 (512.5–672.0)	281.0 (151.6–410.4)

^1^ “None of the above” accounted for 1.4% (*n* = 64) of the sample and “don’t know” for *n* = 2. All analyses are population weighted. ^2^ Data excludes children whose proportion was 50:50, e.g., had 50% full cream and 50% reduced fat (*n* = 32 for plain milk and *n* = 27 for plain and flavoured milk).

Saturated fat content of the total diet, as a percentage of energy, was highest for those who reported whole milk as their main milk type (14.3%) and lowest for those who reported soy milk as their main milk type (10.5%). Saturated fat intake was similar for children who reported their main milk type was low/reduced fat (12.7%) or skim milk (12.6%), and for those who reported they did not consume milk (12.4%). The mean volume of milk consumed (±flavoured milk) decreased as children reported lower fat milk choices as their main milk type by short question (*p* < 0.001). Those who reported they did not consume milk by the short question consumed a daily average of 227 mL in the recalls, with one third consuming some milk (Table 4).

## 4. Discussion

Short survey instruments measuring fruit and vegetable intake typically use between six and 16 questions [13]; however, single item questions to measure fruit intake or vegetable intake have also been used [14,15]. The standard short questions on usual fruit intake, usual vegetable intake and usual milk type used in the 2007 Australian National Children’s Nutrition and Physical Activity Survey showed acceptable validity for groups of Australian children compared to information from 24-h dietary recalls.

As is generally recommended [16], parents were the primary reporters of young children’s intake in this survey, and in these age groups estimation of the mean fruit intake using the short question was similar to estimation using two detailed 24-h dietary recalls. Relatively good validity has also been shown with Australian parents reporting their pre-school aged (2–5 years) children’s usual intake on single item questions for fruit and vegetables compared to three-day food records [17]. In groups aged between nine and 16 years, estimation of fruit intake using the short question was higher by less than half of a serve (75 g) compared to estimation from the recalls. Children reporting high fruit intake by short question (4, 5, or 6+ serves a day) markedly overestimated their fruit intake compared to 24-h recall, however, this was <20% of the sample.

Estimation of mean vegetable intake of groups aged four years and older using the short question was also higher by less than half a serve (75 g) compared to estimation using recalls. Again, children reporting higher vegetable intake by short question substantially overestimated their vegetable intake. A modest decrease in fruit intake with age group was detectable using the 24-h dietary recall data, but not in the data from the short questions. However, an increase in mean vegetable intake with age group was detectable using both methods. While the differences in the group mean estimates of intake between the short questions and 24-h recalls may appear minor, less than one half of a 75 g serve, this represents up to 20%–30% of children’s intake (depending on age).

Consumption data from two 24-h dietary recalls was consistent with the response provided by short question regarding the main type of milk usually used. Agreement between methods was similar when frequency rather than volume of milk consumption was used, but was slightly lower when flavoured milk was included. The reason for this maybe because children who consume flavoured milk tend to consume whole flavoured milk (79.4% of flavoured milk consumers) regardless of the type of milk they report as their main milk type. Importantly, milk type used also appeared to be related to milk volume consumed. There was an association between main type of milk and the percentage of total dietary energy contributed by saturated fat, with the small number of children who respond soy milk (~3%) having the lowest percentage energy from saturated fat and the large number of children (~65%) who respond whole milk having the highest percentage energy from saturated fat. Usual type of milk reported has also been shown to relate to saturated fat intake in adults [1].

The short questions about usual fruit and vegetable intake required a response in “serves”, and a reference serve size in a household measure (half a cup in each case) was provided. The reference serve size only provides a guide because fruit and vegetables are not necessarily eaten or easily conceived in “half cup” multiples. Furthermore, portion size differs across age groups. We considered whether subjects responded after taking into account the reference serve size provided, or responded according to the number of occasions (frequency) they usually consumed a food. For fruit intake, the mean frequency of fruit intake by 24-h recall for each short question response category was substantially less than the reported intake of 75 g serves from the short question, but rather paralleled the intake of 150 g serves. We interpret this as evidence that the average serve size of fruit consumed by children was close to 150 g but that subjects provided a response to the short question that incorporated the reference serve size provided in the question. Researchers in the US have shown that the inclusion of a reference serve size description in a short question makes a large difference to the group response given [18].

Other state-based Australian surveys [15,19,20,21] have used the recommended serve size in the Australian Guide to Healthy Eating [10], however, short questions with a half cup reference serve size have also been used internationally [18,22]. For Australian children, the mean consumed portion size of fruits varies from 92 g to 141 g, and for vegetables from 41 g to 80 g for 2–3 year olds and 14–16 year olds, respectively [3]. Selection of a reference size in a single question is important but will not be consistent with consumed portion size for all age groups.

The short questions about fruit and vegetable intake did not specify what foods to include. For the present analysis, potato crisps and other fried potato foods were excluded from the vegetable group for estimating intake from the 24-h recall data; however, some subjects may have included these foods in their short question response. Data from the 24-h recalls suggests that vegetable intake increased with age, and the discrepancy between the estimates from the recalls and the response on the short question increased with intake. As a single category, potato accounts for 18%–22% of children’s total vegetable consumption depending on age, and up to 40% if fried potato products are included. Also, the amount of potato reported per eating occasion is almost double that of other vegetables whereas for other vegetables it is less than one serve (110 g *vs*. 65 g on average across all age groups). So, as total vegetable intake increases, potatoes account for a relatively smaller proportion of total intake. This may partially explain the increasing discrepancy between methods as intake increases.

When 100% fruit juice was included into the estimation of total fruit intake by dietary recall, we found that a similar amount was consumed regardless of the short fruit question response given. Furthermore, inclusion of 100% fruit juice implied a substantial under estimation of fruit intake by the short question. Subjects probably do not include fruit juice when answering the current question. The Australian Guide to Healthy Eating states that half a cup of 100% fruit juice is an alternative to one piece of fresh fruit [10], therefore measuring intake is of interest. However, inclusion of 100% fruit juice in a short question is not necessarily straightforward, with a study in U.S. adults suggesting that the term “100% fruit juice” was not appropriately understood and adults included fruit drink in this category, inflating their estimated fruit intake [23]. Further work is required to develop either a single short question to include 100% fruit juice in fruit intake, or to develop a separate question about 100% fruit juice intake.

Almost one quarter of Australian children are overweight or obese [24,25], so it is relevant to understand how weight status might potentially influence dietary intake estimates in population surveys. While fruit intake was higher by short question than by 24-h recall for all children, for obese children the difference was more than twice as much as normal weight or overweight children. Children with higher BMIs are more likely to under report their energy intake in 24-h recalls compared to individuals with lower BMIs [26], and also parents of obese children are more likely to under report their children’s intake more than parents of normal weight children [27]. In this analysis, the larger difference between methods in fruit intake for obese children may actually be due to a more accurate estimate of fruit intake by short question. However, a Canadian study [28] using a six item questionnaire to measure fruit and vegetable intake over the previous seven days found little difference between obese and non-obese individuals, and there was no difference by weight status in short question performance for vegetable intake. Other individual characteristics not explored in this analysis may also influence the performance of short dietary questions. For example, a short question about fruit intake has been shown to perform better in non-Indigenous Australian children than in Aboriginal and Torres Strait Islander children. The question was able to detect an increasing trend in intake (relative to three dietary recalls) in the non-Indigenous children; however, this trend was not evident in the Aboriginal and Torres Strait Islander children [29].

Type of milk used was dominated by children who stated they usually had whole milk (~65%). More than 80% of these children did have mostly whole milk by volume, and most often had whole milk by frequency, based on two days of dietary recall. This underestimates the true level of agreement because two days of recall does not necessarily confirm the main type of milk used. In particular, between 10% and 19% of children could not be assigned a “main type of milk” because they did not consume milk on the days of the dietary recall. The small percentage of children (~5%) who responded that their usual type of milk was skim milk were less likely to have consumed milk, and almost a third mostly consumed another type of milk on the days of recall. A small percentage of children (<2%) reported that they did not use milk; however, about one third of these were found to consume milk by dietary recall. Milk has many different dietary usages and respondents may only consider a particular milk usage in their response to the short question.

Short dietary questions are useful in monitoring population intakes when they accurately reflect true differences or changes in intake between relevant population groups. Responses to single short questions on fruit and vegetable intake were compared directly to food intake from recalls and not nutrient intakes (sometimes referred to as indirect validity). Responses on short questions on fruit and vegetables have also been shown to correlate to biomarkers such as serum B-carotene, red cell folate [30,31], serum vitamin C and urinary potassium [30] for fruit intake, and serum B-carotene and red cell folate for vegetable intake [31] in Australian and UK adult samples, which provides support for their validity. Ideally short questions would accurately reflect true intake differences between relevant population groups. In our analyses, a short question on fruit intake was not sensitive enough to show the small decrease in fruit intake with age.

This study utilises data from a nationally representative sample of Australian children where response to short dietary questions was collected prior to collection of two 24-h dietary recalls. The fruit intake estimate would benefit by the inclusion of a further question on intake of 100% fruit juice (which is not captured by the current question).

## 5. Conclusions

Even single short questions provide reasonable estimates of fruit and vegetable intake compared to detailed 24-h dietary recalls. A short question about milk type is a crude surrogate for percentage of total dietary energy contributed by saturated fat. Ease of administration and data processing suggest that short dietary questions, at least for some aspects of dietary intake, are a useful method to monitor population dietary intake of children, and to assess the impact of programs between the administrations of more detailed surveys.

### Implications

- At the group level, single questions provide reasonable estimates of children’s fruit and vegetable intake compared to detailed 24-h dietary recalls, and milk type is a crude surrogate for percentage of total dietary energy contributed by saturated fat.
- The accuracy of reporting intake using a single question can vary by children’s age, gender and weight status. In addition, the wording of questions to measure intake can also vary between surveys. These variations need to be considered in the interpretation of eating habits data from population surveys using single questions.
